# Eukaryotic LYR Proteins Interact with Mitochondrial Protein Complexes

**DOI:** 10.3390/biology4010133

**Published:** 2015-02-12

**Authors:** Heike Angerer

**Affiliations:** Goethe University Frankfurt, Medical School, Institute of Biochemistry II, Structural Bioenergetics Group, Max-von-Laue Street 9, Frankfurt am Main 60438, Germany; E-Mail: Angerer@em.uni-frankfurt.de; Tel.: +49-69-798-29575

**Keywords:** LYR proteins, LYRM, LYR motif, mitochondria, OXPHOS complexes, respiratory complex I, mitochondrial acyl-carrier protein, ACPM, mitochondrial fatty acid synthesis type II, lipoic acid (6,8-dithio-octanoic acid), reactive oxygen species, insulin resistance

## Abstract

In eukaryotic cells, mitochondria host ancient essential bioenergetic and biosynthetic pathways. LYR (leucine/tyrosine/arginine) motif proteins (LYRMs) of the Complex1_LYR-like superfamily interact with protein complexes of bacterial origin. Many LYR proteins function as extra subunits (LYRM3 and LYRM6) or novel assembly factors (LYRM7, LYRM8, ACN9 and FMC1) of the oxidative phosphorylation (OXPHOS) core complexes. Structural insights into complex I accessory subunits LYRM6 and LYRM3 have been provided by analyses of EM and X-ray structures of complex I from bovine and the yeast *Yarrowia lipolytica*, respectively*.* Combined structural and biochemical studies revealed that LYRM6 resides at the matrix arm close to the ubiquinone reduction site. For LYRM3, a position at the distal proton-pumping membrane arm facing the matrix space is suggested. Both LYRMs are supposed to anchor an acyl-carrier protein (ACPM) independently to complex I. The function of this duplicated protein interaction of ACPM with respiratory complex I is still unknown. Analysis of protein-protein interaction screens, genetic analyses and predicted multi-domain LYRMs offer further clues on an interaction network and adaptor-like function of LYR proteins in mitochondria.

## 1. The LYR Motif

**Figure 1:**
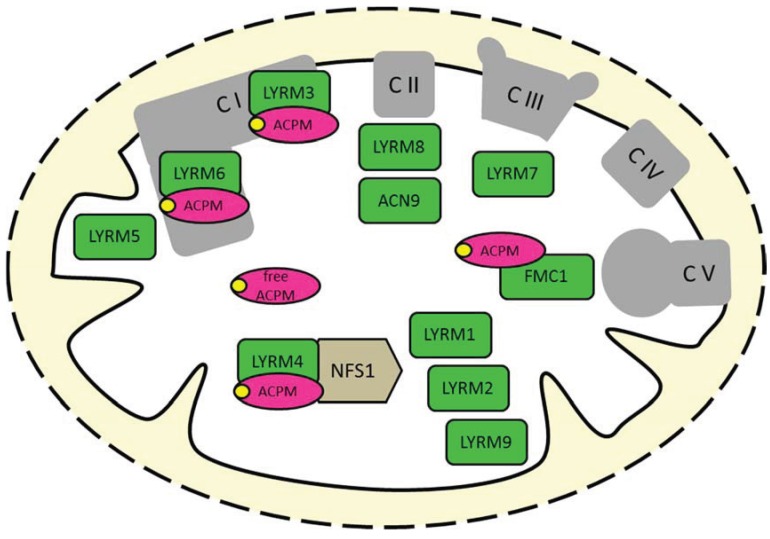
LYRMs in mitochondria of (higher) eukaryotes. Many LYRMs (green) are subunits or assembly factors of OXPHOS complexes. Four LYRMs interact with the mitochondrial acyl carrier protein (ACPM/NDUFAB1, magenta), which is also a duplicated subunit of complex I [23,24]. LYRM4 is an essential protein of the Fe-S cluster biogenesis machinery. LYRM5 is a complex I-related protein (see the main text). The function of LYRM1, LYRM2 and LYRM9 in mitochondria is unknown. C, complex; NFS1, cysteine desulfurase; yellow circles, phosphopantetheine cofactor of ACPM.

The LYR proteins (LYRMs) of the Complex1_LYR-like superfamily are exclusively found in eukaryotes. The Complex1_LYR-like superfamily (Pfam clan CL0491) was manually curated by P. Coggill from the Wellcome Trust Sanger Institute/European Bioinformatics Institute (EMBL-EBI) [1], and it has three Pfam-A members: the LYR-, LYR_1- and LYR_2-protein family. LYRMs are basic ~15 kDa polypeptides and carry a conserved tripeptide L-Y-R (leucine/tyrosine/arginine) sequence close to the N-terminus (Table 1). Furthermore, some basic arginine/lysine residues and a highly-conserved phenylalanine are downstream elements of the LYR motif in the LYR- and LYR_1-protein family, respectively. The LYR_2 motif is characterized by the N-terminal L-Y-R sequence and a pattern of tyrosines/glycine at the C-terminus (reviewed in [2]). The human genome contains at least ten LYRMs that were predominantly identified as mitochondrial proteins (Figure 1) [3,4,5,6]; however, some members of this protein superfamily were also found in the cytosol or nucleus [7,8]. Human LYRMs are linked with diseases, such as insulin-resistance (LYRM1 [9]), muscular hypotonia (LYRM3 [10]), deficiency of multiple OXPHOS complexes (LYRM4 [11]), apoptosis in HIV-1 infection (LYRM6 [12]), encephalopathy and lactic acidosis (LYRM7/MZM1L [13]), infantile leukoencephalopathy (LYRM8/succinate dehydrogenase assembly factor (SDHAF) 1 [14]) and alcohol dependence (acetate non-utilizing protein (ACN) 9/SDHAF3 [15]). In this review, the human nomenclature of proteins is used throughout; otherwise, the nomenclature of the respective organism is indicated.

The original classification of LYRMs was based on LYRM4, which interacts with the cysteine desulfurase NFS1, a central component of the ancient Fe-S cluster biogenesis machinery [16,17]. Accordingly, there was an initial bias to assume a role in mitochondrial Fe-S cluster biogenesis for all LYRMs, e.g., prompted by the Pfam database (www.pfam.xpfam.org/clan/CL0491). However, LYRM4 seems to represent the only LYR protein that is directly involved in the first steps of Fe-S cluster generation. Furthermore, other LYRMs have been identified as accessory subunits or assembly factors of mitochondrial OXPHOS complexes I, II, III and V, respectively, and to play a role in acetate metabolism [2]. Nevertheless, a common specific physiological role of LYRMs and the structural role of the LYR motif are still unknown.

The tripeptide sequence L-Y-R is a central part of the LYR motif in LYRMs, but the entire motif requires further conserved amino acid residues (Table 1). However, it should be noted that the term “LYR proteins” has been used by some authors to classify proteins with an L-Y-R tripeptide, but lacking other features of the LYR motif. The sequence stretch L-Y-R is frequently found in mitochondrial proteins, e.g., in the sequence of complex I assembly factor NDUFAF3, complex II subunit SDHB, complex IV subunits 4 and 7A, complex V subunit B1 and ribosomal proteins (Supplemental Figure S1); however, these proteins are not LYRMs. Recently, the L-Y-R sequence was suggested to present an interaction site for mitochondrial co-chaperone binding [18]. The DNAJ-type III co-chaperone HSC20 was described as a component of the ISCU/HSPA9 protein complex that transfers Fe-S clusters from the scaffold protein ISCU to target proteins [19,20]. Complex II assembly factor LYRM8/SDHAF1, complex II subunit SDHB and complex III assembly factor LYRM7/MZM1L carry L-Y-R(-like) sequences (Table 1, Supplemental Figure S1). In co-immunoprecipitation and yeast two hybrid experiments, LYRM7, LYRM8 and SDHB were shown to interact with the co-chaperone HSC20 via the L-Y-R(-like) sequences [18,20]. However, subunit SDHB is not defined as LYRM, as analyzed using the bioinformatics tool Pfam search (www.pfam.xfam.org/search).

**Table 1:** Human LYRMs in mitochondria. The theoretical molecular masses of LYRMs range between 10 kDa and 22 kDa and the theoretical pI between nine and 11. LYRMs are predominantly mitochondrial proteins; however, some LYRMs reside also in the cytoplasm or nucleus, as reported for LYRM1 and LYRM4, respectively [7,8]. The most important conserved residues of LYRMs are shown in the sequence alignment of the human proteins (highlighted in red). C, complex; ISC, iron sulfur cluster; COPP, complex I phylogenetic profile; AF, assembly factor.

Human Protein	m (kDa)	pI	Mitochondrial Localization	Import Probability [21]	Section of Sequence Alignment ^a^ (Numbers Indicate Amino Acid Residues) [22]	Function	Interaction Partner
LYRM1	14	10	Yes [3]	93	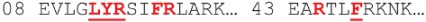	insulin signaling	unknown
LYRM2	11	11	Yes [3]	99	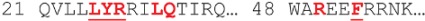	unknown	unknown
LYRM3/NDUFB9	22	9	Yes [3]	90	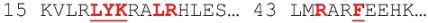	CI subunit	ACPM
LYRM4/ISD11	11	11	Yes [3]	92	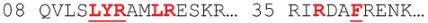	ISC biogenesis	NFS1, ACPM
LYRM5	11	10	Yes [3]	35	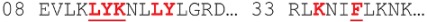	COPP	unknown
LYRM6/NDUFA6	18	10	Yes [3]	94	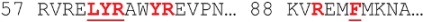	CI subunit	NDUFS3, ACPM
LYRM7/MZM1L	12	10	Yes [3]	87	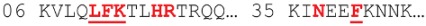	CIII AF	UQCRFS1, HSC20
LYRM8/SDHAF1, yeast Sdh6	13	11	Yes [14]	29	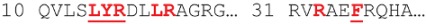	CII AF	SDHB, HSC20
LYRM9	10	9	unknown	99	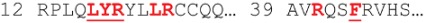	unknown	unknown
ACN9/SDHAF3, yeast Sdh7	15	9	Yes [4,5]	98	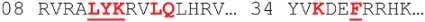	CII AF	SDHB
FMC1/C7orf55 ^b^	13	10	Yes [6]	90	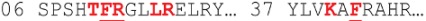	CV AF	ATP12, ACPM

^a^ ClustalW2, default parameters; the sequence of LYRM6 was manually shifted; ^b^ formation of mitochondrial complexes protein 1, not a LYRM, as analyzed using the Pfam search tool (see the main text).

**Figure 2:**
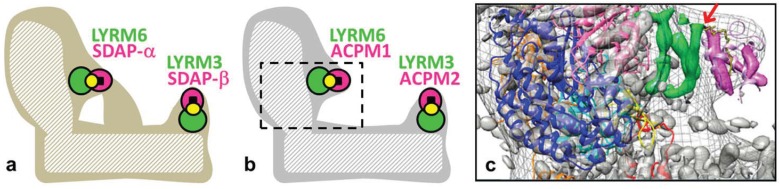
LYRM subunits of mitochondrial complex I. (**a**) Schematic model of complex I from *Bos taurus* after [24]. The dashed area represents the central subunits harboring the catalytic core functions. Accessory subunits (filled areas) form a scaffold around the central subunits. Two accessory domains, one at the peripheral matrix arm and one at the distal proton pumping membrane arm, are formed by LYRMs (green). LYRM6 and LYRM3 interact with the mitochondrial acyl-carrier protein subunit (ACPM, bovine SDAP, magenta) that carries phosphopantetheine cofactors (yellow circles) and, additionally, acyl groups (black squares). (**b**) Schematic model of *Yarrowia lipolytica* complex I in analogy to bovine complex I [23]. The frame in (**b**) indicates the zoom window of the structural modeling of subunits LYRM6 and ACPM1 in (**c**) [25]. (**c**) Overlay of X-ray electron density map (gray surface, contour level 1.9) with an EM envelope of *Yarrowia lipolytica* complex I (gray mesh) and the X-ray structure of bacterial *Thermus thermophilus* complex I (PDB file 4HEA); central subunits: NDUFS2 subunit (NQO4, blue; N-terminal β-sheet highlighted in yellow), NDUFS7 subunit (NQO6, cyan), NDUFS8 subunit (NQO9, orange), NDUFS3 subunit (NQO5, hot pink), loop connecting helices 1 and 2 of subunit ND3 (NQO7, red); structure of ACP from *E. coli* (PDB file 2FAE, pink; decanoyl phosphopantetheine, yellow stick representation) fitted to electron density (magenta); please compare the original figure of the publication [25]. Electron density features tentatively assigned to subunit LYRM6 are highlighted in green. The red arrow indicates the strong contact between LYRM6 and the modelled phosphate group of the phosphopantetheine cofactor from ACPM1.

The downstream phenylalanine of the LYR motif (Table 1) might play an important role in the function of LYRMs. Complex I accessory subunit LYRM6/NDUFA6 (yeast NB4M) from the yeast *Yarrowia lipolytica* is essential for complex I activity and anchors a mitochondrial acyl-carrier protein ACPM/NDUFAB1-α (yeast ACPM1) to complex I [25] (Figure 2b). Mutation of the L-Y-R sequence of LYRM6 resulted in slightly reduced abundance of complex I in mitochondrial membranes with no effect on normalized complex I activity. A much more drastic effect was observed only after mutation of the L-Y-R sequence in conjunction with the conserved downstream phenylalanine (LYR + F). Subunit ACPM1 was detached from the enzyme complex, and complex I activity was abolished. Thus, the LYR motif of subunit LYRM6 might be important to bind the ACPM subunit. Complexome profiling of intact mitochondria showed that LYRM6 co-exists as complex I-associated and in free form in the matrix space [25]. It cannot be excluded that the soluble matrix LYRM6 might have extra functions, e.g., as a maturation factor of the Fe-S cluster containing central subunits increasing the abundance of complex I in mitochondrial membranes. Taken together, LYRMs are basic hydrophilic proteins interacting with mitochondrial macromolecular complexes, such as the OXPHOS complexes and the NFS1/ISCU complex of Fe-S cluster biogenesis. There are indications that LYRMs are adaptor-like mitochondrial factors that promote the assembly and function of multi-subunit protein complexes.

## 2. The Diverse Roles of the LYR Motif Proteins in Mitochondria

### 2.1. Complex I Subunits LYRM3, LYRM6 and ACPM

Mitochondrial complex I (NADH:ubiquinone oxidoreductase) is an L-shaped membrane protein with a hydrophilic peripheral arm and a hydrophobic membrane arm [26,27]. Interestingly, yeast and mammalian complex I contain two LYRMs, LYRM6/NDUFA6 (yeast NB4M, bovine B14) and LYRM3/NDUFB9 (yeast NI2M, bovine B22), and two versions/copies of the acyl-carrier protein subunit ACPM/NDUFAB1-α/β (yeast ACPM1/2, bovine SDAP-α/β) [24,28,29]. The basic LYRM3 and LYRM6 (pI = 9 and 10) might bind the acidic ACPM (pI without phosphopantetheine cofactor = 4) to complex I via strong polar interactions, forming essential accessory domains. Based on structural and biochemical characterization of complex I from the *lyrm*6Δ deletion strain, the LYRM6/ACPM1 domain is located at the matrix arm close to the ubiquinone reduction site (Q module). In HIV-1 infected T-cells, the gene of *LYRM6* is downregulated, and the activity of complex I is impaired, resulting in the induction of the apoptotic pathway [12]. The deletion studies in the model organism *Yarrowia lipolytica* suggested that the downregulation of human *LYRM6* in HIV-infected T-cells leads to the loss of the LYRM6/ACPM-α domain of human complex I that is essential for catalytic activity and finally results in cell death. Subunit LYRM3 has been shown biochemically to reside at the distal part of the membrane arm (proton pumping P_D_-module) [30]. In analogy to LYRM6/ACPM1, LYRM3 and ACPM2 together might form a common “duplicated” accessory domain of mitochondrial complex I (Figure 2). In fact, in the EM structure of bovine complex I, the SDAP-β subunit (ACPM2) was identified at the tip of an accessory domain at the matrix-faced part of the P_D_-module [24]. Thus, the accessory domain LYRM6/ACPM-α has been described for the yeast and bovine complex; and the LYRM3/ACPM-β domain has been proposed for the bovine enzyme [23,24]. Deletion studies of ACPM1 and ACPM2 in *Yarrowia lipolytica* revealed that ACPM1 is an essential protein for the yeast, while ACPM2 is essential for complex I assembly/stability [31]. Downregulation of human ACPM compromises protein lipoylation, abolishes specific complex I activity and ultimately results in cell death [32]. ACPM is an essential protein of the mitochondrial fatty acid synthesis type II that forms octanoyl-ACPM precursors for the biosynthesis of lipoic acid (6,8-dithio-octanoic acid) [33] and of organelle-specific lipids [34]. In general, lipoylation of mitochondrial α-keto dehydrogenases is indispensable for mitochondrial metabolism, and lipoic acid is not a vitamin that can be supplied externally [32,33,35,36]. Summarized, the LYRM containing domains LYRM6/ACPM-α and LYRM3/ACPM-β play important roles in the activity and assembly/stability of complex I from yeast to human.

So far, only limited structural information is available about LYRMs. Prediction of secondary structures using the bioinformatics toolkit Quick2D (www.toolkit.tuebingen.mpg.de/quick2_d) suggested that they are small mainly α-helical hydrophilic proteins. The molecular weight of mitochondrial complex I is almost 1 MDa [27,37,38], and the LYRM6/ACPM domain (bovine B14/SDAP-α) represents just 3% of the total protein mass [30,37]. The loss of this domain by chromosomal deletion of *LYRM6* had no impact on the EPR signals of the (NADH reduced) Fe-S clusters of subcomplex LYRM6Δ, except for a minor effect on the signal intensity of Fe-S cluster N2 from the central subunit NDUFS7 (bovine PSST) [25]. Thus, the terminal Fe-S cluster N2, which is part of the ubiquinone reduction site, can be reduced and presents electrons to ubiquinone molecules. However, as mentioned above, subcomplex LYRM6Δ is enzymatically inactive. Figure 2 shows the simplified structural models of mitochondrial complex I from bovine and *Yarrowia lipolytica* (Figure 2a,b, respectively). A tentative modeling of subunits LYRM6 and ACPM1 into the X-ray crystallographic map of *Yarrowia lipolytica* complex I (Figure 2c, green and magenta, respectively) provides structural insights into this domain [25]. Similar as reported for the bovine enzyme [24], LYRM6 is comprised of three α-helices (Figure 2c, green) and interacts with the central subunits NDUFS3 and NDUFS2 (bovine 30 kDa and 49 kDa) and on the distal side with ACPM1 (Figure 2c, magenta). Thus, ACPM1 contacts complex I exclusively via LYRM6 close to the ubiquinone reduction site. The structural analysis also suggests that the exposed negatively-charged phosphate moiety of the phosphopantetheine cofactor of ACPM1 (Figure 2c, yellow stick representation; see the red arrow) is critically involved in the binding to LYRM6. Interestingly, the contact site between LYRM6 and the central NDUFS2 subunit is a structural element that is critical for the catalytic activity of mitochondrial complex I [39]. The interaction of ACPM with this structural element might regulate complex I activity by so far unknown factors. Complex I subunit ACPM carries a long-chain acyl residue in its binding pocket [34,37,40] that might function as one of these regulatory factors.

**Figure 3:**
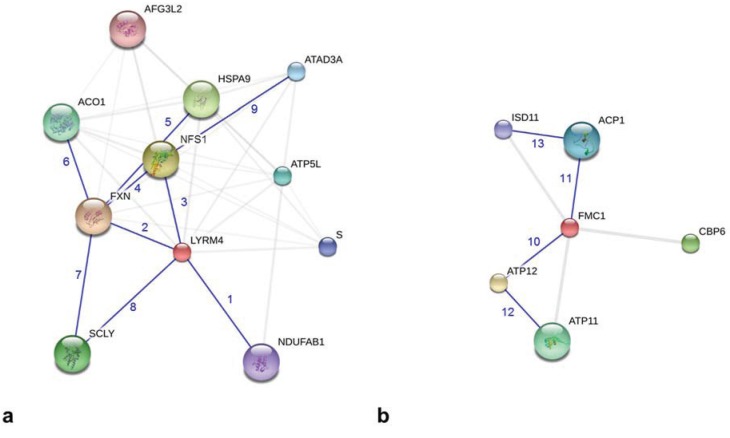
Graphical description of protein-protein interactions of human LYRM4 and FMC1 from *S. cerevisiae* using the bioinformatics tool STRING (modified actions views; [41]). (**a**) LYRM4 (red colored sphere) interacts with the enzymes of Fe-S cluster biogenesis, NFS1 and FXN. In addition, the interactions with selenocysteine lyase (SCLY) and ACPM (NDUFAB1) are shown. (**b**) FMC1 (red colored sphere) interacts with complex V assembly factor ATP12 and with ACPM (ACP1). Blue numbers indicate the experimentally shown protein-protein interactions (relevant information transferred from other species): 1, LYRM4-NDUFAB1 [43]; 2, LYRM4-FXN [45]; 3, LYRM4-NFS1 [43]; 4, FXN-NFS1 [52]; 5, FXN-HSPA9 (heat shock 70-kDa protein 9) [45]; 6, FXN-ACO1 (aconitase 1) [53,54]; 7, FXN-SCLY (by homology to NFS1); 8, LYRM4-SCLY (by homology to NFS1); 9, NFS1-ATAD3A (ATPase, AAA containing domain 3A) [43]; 10, FMC1-ATP12 [42]; 11, FMC1-ACP1 [42]; 12, ATP12-ATP11 [42]; 13, ACP1-ISD11 [42]. AFG3L2, AFG3 ATPase family gene 3-like 2; ATP5L, ATP synthase subunit G; S, cDNA FLJ78714; CBP6, cytochrome B pre-mRNA-processing protein 6.

The duplicated interaction of respiratory complex I with ACPM seems to represent an essential protein-protein interaction that needs two adaptor LYRMs. Analyses of other LYRMs using the protein-protein interaction database STRING provides further insights into their functional protein association networks [41]. As expected, e.g., human LYRM4 interacts with the proteins of Fe-S cluster biogenesis, such as NFS1 and FXN, and complex V assembly factor FMC1 (LYR_2 motif) from *Saccharomyces cerevisiae* forms a complex with the assembly factor ATP12 (Figure 3a,b). In addition, the STRING analysis shows that LYRM4 and FMC1 also interact with ACPM [42,43]. Accordingly, I suggest that the structure of the LYR motif might present a general adaptor for interaction with the ACPM protein. The exposed negatively-charged phosphate moiety of ACPM’s cofactor might play an important role in the structural stabilization of the protein-protein interaction between ACPM and LYRMs(Figure 2c, [25]).

### 2.2. Essential Factor of Fe-S Cluster Biogenesis (LYRM4)

The biosynthesis of Fe-S clusters in the matrix of mitochondria is an essential pathway of eukaryotic cells [17,20,44]. LYRM4 (ISD11) has been shown to interact with the ancient Fe-S cluster biogenesis components NFS1 (cysteine desulfurase), the scaffold protein ISCU and frataxin (FXN, Friedreich ataxia protein) [16,45,46]. The LYRM4/NFS1/FXN complex has been suggested to provide the Fe and S atoms for the Fe-S cluster assembly occurring at the ISCU scaffold proteins. The transfer of Fe-S clusters to the target proteins is performed by the ISCU/HSPA9/HSC20 complex [20]. Depletion of LYRM4 by siRNA in human HEK293T cells resulted in an increased intracellular Fe content, as is observed in Friedreich’s ataxia, caused by defects in FXN [45]. Mutations of the L-Y-R sequence in LYRM4 from *Saccharomyces cerevisiae* resulted in partial loss of Nfs1 function [47]. Recently, LYRM4 was shown to influence the oligomeric state of NFS1 [48]. Interestingly, specific interactions of the lipid phosphatidylinositol 3,5-biphosphate (PIP2, substrate of PI3K) with LYRM4 were shown in profiles of lipid-protein interactions from *Saccharomyces cerevisiae* [49].

The mitochondrial production of Fe-S clusters is essential to generate Fe-S cluster-containing proteins. Furthermore, the availability of 4Fe-4S clusters (sulfur donors) and octanoyl-ACPM are both essential for the production of lipoic acid. Lipoic acid synthase (LIAS) exploits 4Fe-4S clusters to transfer two sulfur atoms to octanoyl-loaded target proteins, forming the lipoylated protein products [50,51]. In this context the protein-protein interaction of (octanoyl-)ACPM with the NFS1/LYRM4 complex might make sense (Figure 3a).

### 2.3. Complex I-Related LYRM5

LYRM5 is a complex 1 phylogenetic profile (COPP) protein [3]. COPPs are absent in organisms that do not contain respiratory complex I. Accordingly, many complex I assembly factors are COPPs [55]. Enzymes involved in the metabolism of branched-chain amino acids (BCAA) and branched-chain fatty acids (BCFA) are also COPPs. BCAA and lipids were suggested to play a central role in the development of insulin resistance in diabetes [56]. Leucine regulates the activation of the mammalian target of rapamycin (mTOR) complex 1, which results in uncoupling of insulin signaling [57]. In *Caenorhabditis elegans*, a specific BCFA is involved in the post-embryonic growth control in parallel with the insulin receptor signaling pathway [58,59]. Furthermore, a BCFA induces mitochondrial-mediated apoptosis in human bladder cancer cells by regulation of the Akt/PKB and MAPK phosphorylation pathways [60]. Interestingly, the mitochondrial metabolism of BCAA by the branched chain α-keto dehydrogenase (cofactor lipoic acid) and the biosynthesis of organelle-specific lipids (fatty acids) both depend on the biosynthetic function of complex I subunit ACPM/NDUFAB1 that produces mitochondrial acyl chains *de novo* [61].

### 2.4. Assembly Factors of OXPHOS Complexes (LYRM7, LYRM8, ACN9, FMC1)

The assembly of succinate:ubiquinone oxidoreductase (SDH, complex II) subunit SDHB, which contains three Fe-S clusters [62], is mediated by the two LYRM assembly factors, LYRM8/SDHAF1 and ACN9/SDHAF3 [5,18]. The LYRMs assist the maturation of SDHB and probably protect the transferred Fe-S clusters from oxidative stress [5]. Both LYRMs improve the production of complex II and increase mitochondrial complex II abundance. Accordingly, pathogenic mutations in LYRM8 induce SDH-defective infantile leukoencephalopathy [14]. Both LYRMs do not take part in the direct insertion of the Fe-S clusters into subunit SDHB. However, the binding of HSC20 to SDHB and LYRM8 guides Fe-S cluster delivery and likely assists Fe-S cluster insertion [18,20]. LYRM8/SDHAF1 and ACN9/SDHAF3 were suggested to protect SDHB from oxidative stress during the assembly process [5]. High concentrations of reactive oxygen species (ROS) damage Fe-S clusters, and protection of the sensitive Fe-S clusters is important in the presence of oxidative stress. Complex I and III produce a significant amount of ROS, and as shown recently, complex II is also discussed to play a role in mitochondrial ROS generation [63].

The dimer of ubiquinol:cytochrome *c* oxidoreductase (*bc*_1_ complex, complex III) contains two Fe-S cluster-containing Rieske proteins (UQCRFS1) [64]. LYRM7 has been described as an accessory factor that stabilizes the Rieske protein prior to assembly to complex III without a chaperone-like function for insertion of the Fe-S cluster [65,66]. Recently, the same function was shown for the human LYRM7/MZM1L protein [67]. Mutation of the tyrosine in the L-Y-R sequence of complex III assembly factor LYRM7/Mzm1 from *Saccharomyces cerevisiae* resulted in the decrease of functional complex III [65], and human pathogenic mutation D25N in LYRM7/MZM1L induces the severe reduction of complex III activity [13]. Similar to LYRM8 and SDHB, LYRM7 interacts with the co-chaperone HSC20 [18]. This protein-protein interaction might protect the Rieske subunit from oxidative stress and support the stability until the protein is inserted into the membrane.

Human formation of mitochondrial complexes protein 1 (FMC1, Table 1) is not recognized as LYRM by the Pfam search tool; however, other mammalian and yeast homologs are members of the LYR_2 family. Mitochondrial ATP synthase (complex V) assembly factor FMC1 interacts with ATP12 [42], which plays a central role in the assembly of the F_1_ component of ATP synthase [6] (Figure 3b).

### 2.5. LYRM1, LYRM2 and LYRM9

The gene of LYRM1 is highly expressed in omental adipose tissue of obese individuals and is an obesity-related gene involved in adipocyte differentiation and growth [7,68,69]. The overexpression of LYRM1 in mature 3T3-L1 adipocytes resulted in reduced insulin-stimulated uptake of glucose and impaired mitochondrial function [70]. High ROS levels were observed that were suggested to trigger the development of mitochondrial dysfunction and insulin-resistance. The knock-down of LYRM1 in these cells resulted in improved mitochondrial function [9] and even rescued the destructive effects of the uncoupling compound, carbonyl cyanide-4-(trifluoromethoxy)-phenylhydrazone (FCCP) [71]. Interestingly, the supply of lipoic acid had a positive effect on insulin-dependent signaling in L6 myotubes [72] and ameliorated the negative effects induced by LYRM1 overexpression [73]. A similar outcome was observed for the anti-diabetic drug, Metformin [74], which is also an inhibitor of respiratory complex I [75]. Overall, these results suggest that LYRM1 takes part in a metabolic redox-dependent signaling pathway connected to mitochondrial biogenesis and homeostasis (PI3K, Akt/PKB pathway).

LYRM2 is the least understood LYRM. There are indications that this protein of higher eukaryotes is involved in DNA repair [76].

The *LYRM9* gene (C17orf108) has been identified as one of four response biomarkers for the immunosuppressive compound, MPA (mycophenolic acid), which is commonly used to treat patients with solid organ transplants [77]. *LYRM9* was also discussed in the context of eosinophilic airway inflammation and particularly in association with childhood asthma [78]. The fraction of exhaled nitric oxide values (Feno) was related to specific asthma phenotypes. Single-nucleotide polymorphisms (SNP) in the genes of inducible NO synthase *NOS2* and *LYRM9* were suggested to affect Feno values.

## 3. Multi-Domain Organization of LYRMs

Supplementary information about the function of LYRMs might be provided by the analysis of the Pfam domain organization of several predicted, though unreviewed, proteins from the UniProt database [1]. The domain organization of the Pfam clan LYR-like CL0491 predicts 30 LYRMs in protein fusions with other protein domains of different function (http://pfam.xfam.org/clan/LYR-like#tabview=tab1). Many predictions of multi-domain proteins might be wrong simply by errors in protein annotation; however, some constructs might be physiologically reasonable, e.g., the fusion of an LYRM assembly factor with another mitochondrial assembly factor. However, in *Saccharomyces pombe*, it has been demonstrated that the mitochondrial protein, tandem Rsm22-Cox11, does not function as a fused multi-domain protein [79]. In fact, after the import of this polypeptide into mitochondria, the protein domains are cleaved, resulting in matured individual proteins. Nevertheless, Table 2 shows nine selected candidates for fused LYRMs that might occur in the respective organisms. In *Penicillium marneffei* and *Talaromyces stipitatus*, complex II assembly factor LYRM8 is fused to HSP20 in an annotated multi-domain protein (UniProt entries B6QRH6_PENMQ and B8M7E8_TALSN). The protein-protein interaction of LYRM8 and the co-chaperone HSC20 has already been demonstrated [18]. HSP20 is a different chaperone; however, the combination of LYRM8 with a heat shock protein might support its chaperone-like function. The same is possibly true for complex III assembly factor LYRM7, which is fused to the HSP20-like HSP90 binding module (CS domain) in the slime mold, *Dictyostelium fasciculatum* (UniProt Entry F4Q9B8_DICFS). In the Chinese hamster *Cricetulus griseus*, the annotated gene sequence of *LYRM9* is fused to the gene of inducible NO synthase (*NOS2*) (UniProt Entry G3GTQ5_CRIGR). The common functional background of human *LYRM9* and *NOS2* has been studied in genetic analyses of SNPs in the context of airway inflammation [78]. In addition, complex I assembly factor IND1/NUBPL has been suggested to play a role in the mitochondrial translation process [80,81,82]. The fusion of complex II assembly factor ACN9/SDHAF3 with a ribosomal processing protein (RRP7) in the yeast *Pichia pastoris* (UniProt Entry F2QP71_PICP7) might reflect a similar situation for complex II. LYRM1 is discussed to represent a factor in the insulin signaling pathway. In *Salpingoeca* sp., LYRM1 is fused to the mitochondrial small GTPase RAB8 (UniProt Entry F2USF2_SALS5). The fusion of LYRM1 with a Ras-like protein seems to present an interesting overlap of signal transduction pathways. Furthermore, FCM1 is fused to a RAP guanine nucleotide exchange factor (RAP-GEF) in three fungi (UniProt Entries B8MGM3_TALSN, Q5BG81_EMENI and A2Q880_ASPNC). In these organisms, the assembly of the F_1_ fragment from complex V might be connected to the RAP signaling pathway. In five insect species, LYRM4 is fused to the mitochondrial translational releasing factor RF1 (UniProt Entries B4GAC8_DROPE, B4MJX9_DROWI, Q291R9_DROPS, E3XFW0_ANODA and E9IC99_SOLIN). Thus, the biogenesis of Fe-S clusters might be directly coupled to the production of the mitochondrially-encoded subunits of OXPHOS complexes. Overall, regarding the finding that mitochondrial protein tandems are cleaved from each other, the fusion of LYRMs to other protein domains might at least increase the import efficiency of the LYRM constructs and/or coordinate the expression levels of the fused domains, as suggested for Cox11 and Rsm22 in *S. pombe* [79].

## 4. Conclusions

Nuclear-encoded LYRMs of eukaryotes are primarily mitochondrial proteins. In many cases, they function as mitochondrial adaptor proteins and are accessory factors (LYR factors) controlling mitochondrial homeostasis. Mitochondria are the central organelles for efficient ATP production and also play important roles in the induction of apoptosis, Ca^2+^ homeostasis, regulation of cell cycle and Fe-S cluster biogenesis. The assembly and full functionality of mitochondrial OXPHOS complexes depends on intact LYRMs. It is tempting to speculate that the specialization of mitochondria required new factors for the efficient assembly of OXPHOS complexes. Furthermore, mitochondrial oxidative stress might have required protective mechanisms, since Fe-S clusters are sensitive to ROS. LYRM8, which interacts with the co-chaperone HSC20 of the ISCU/HSPA9 complex, and ACN9/SDHAF3 protect Fe-S clusters during the transfer to complex II subunit SDHB. Four LYRMs interact with ACPM, which is the central component of the mitochondrial fatty acid synthesis machinery that produces octanoyl-ACPM precursors for the essential endogenous biosynthesis of lipoic acid. The physiological role of the LYRM/ACPM interaction is still unknown; however, the adaptor-like function of LYRMs to bind ACPM is suggested in this review. Overall, LYRMs play multifaceted roles in bioenergetic, biosynthetic and metabolic signaling pathways that promote mitochondrial biogenesis.

**Table 2:** Selection of LYRMs in domain organization with other proteins. Please note that all proteins are unreviewed, predicted proteins of the UniProt database analyzed by the Pfam data base.

LYRM ^a^	Fused Domain	Organism(s)	Source	LYRM Domain, Amino Acid Residue (Start–End)	Human Orthologue	Pfam Domain, Remarks
LYRM1	Ras	*Salpingoeca rosetta*	http://pfam.xfam.org/protein/F2USF2_SALS5	180–230	RAB8B_HUMAN ^b^	PF00071, small GTP binding protein RAB8
LYRM2	BTB-SPRY	*Naegleria gruberi*	http://pfam.xfam.org/protein/D2VKV5_NAEGR	462–524	KBTB3_HUMAN	PF00651/PF00622, BTB/POZ protein
LYRM3	DEAD/DEAH	*Selaginella moellendorffii*	http://pfam.xfam.org/protein/D8TAY1_SELML	22–81	DDX28_HUMAN ^b^	PF00270, RNA helicase, RNA processing
LYRM4	RF-1	*Drosophila persimili* ^c^	http://pfam.xfam.org/protein/B4GAC8_DROPE	7–61	CL065_HUMAN ^b^	PF00472, translational release factor 1, C12orf65
LYRM7	CS (HSP20-like)	*Dictyostelium fasciculatum*	http://pfam.xfam.org/protein/F4Q9B8_DICFS	6–68	─	PF04969, binding module for HSP90
LYRM8	HSP20	*Penicillium marneffei* ^d^	http://pfam.xfam.org/protein/B6QRH6_PENMQ	105–164	CRYAA_HUMAN	PF00011
LYRM9	NO_synthase	*Cricetulus griseus*	http://pfam.xfam.org/protein/G3GTQ5_CRIGR	11–69	NOS2_HUMAN	PF02898, inducible NO synthase
ACN9	RRP7	*Pichia pastoris*	http://pfam.xfam.org/protein/F2QP71_PICP7	10–67	RRP7A_HUMAN	PF12923, ribosomal RNA-processing protein 7
FMC1	Ras-GEF	*Talaromyces stipitatus* ^e^	http://pfam.xfam.org/protein/B8MGM3_TALSN	568–659	RPGF3_HUMAN	PF00617, RAP guanine nucleotide exchange factor 3

^a^ The identity of the LYRM domains was deduced using the bioinformatics tool, Standard Protein BLAST, with default parameters [83]; ^b^ mitochondrial protein; ^c^ also in *Drosophila willistoni* B4MJX9_DROWI, *Drosophila pseudoobscura* Q291R9_DROPS, *Anopheles darling* E3XFW0_ANODA, *Solenopsis invicta* E9IC99_SOLIN; ^d^ also in *Talaromyces stipitatus* B8M7E8_TALSN; ^e^ also in *Aspergillus niger* Q5BG81_EMENI, *Emericella nidulans* A2Q880_ASPNC.

## Supplementary Files

Supplementary File 1
