# Supplementary material for: Eukaryotic LYR Proteins Interact with Mitochondrial Protein Complexes
Source: Biology (Basel). 2015 Feb 12;4(1):133–50. doi: 10.3390/biology4010133 (PMC4381221; doi:10.3390/biology4010133)
Supplement: Supplementary File 1 [file biology-04-00133-s001.pdf]

Supplementary Materials

Figure S1. L-Y-R sequence containing human mitochondrial proteins that are not LYRMs. Complex I assembly factor NDUFAF3, complex IV isoforms of subunits 4 and 7a, complex V subunit B1 (ATP5F1) and the 39S and 28S mitochondrial ribosomal proteins contain L-Y-R sequences. The L-Y-R sequence has been suggested to function as interaction site for the DNAJ type III co-chaperone HSC20 in the Fe-S cluster transfer process, however the listed proteins do not contain Fe-S clusters. In contrast, Fe-S cluster containing subunit SDHB of complex II harbors two L-Y-R(-like) sequences that have been experimentally shown to interact with the HSC20 (see main text).

>NDUFAF3
MATALALRSLYRARPSLRCPPVELPWAPRRGHRLSPADDELYQRTRISLLQREAAQAMYIDSYNSRGF
MINGNRVLGPCALLPHSVVQWNVGSHQDITEDSFSLFWLLEPRIEIVVVGTGDRTERLQSQVLQAMR
QRGIAVEVQDTPNACATFNFLCHEGRVTGAALIPPPGGTSLTSLGQAAQ
>ATP5F1
MLSRVVLSAAATAAPSLKNAAFLGPGVLQATRTFHTGQPHLVPVPPLPEYGGKVRYGLIPEEFFQFLY
PKTGVTGPYVLGTGLILYALSKEIYVISAETFTALSVLGVMVYGIKKYGPFVADFADKLNEQKLAQLE
EAKQASIQHIQNAIDTEKSQQALVQKRHYLFDVQRNNIAMALEVTYRERLYRVYKEVKNRLDYHISV
QNMMRRKEQEHMINWVEKHVVQSISTQQEKETIAKCIADLKLLAKKAQAQPVM
>COX4-1
MLATRVFSLVGKRAISTSVCVRAHESVVKSEDFSLPAYMDRRDHPLPEVAHVKHLSASQKALKEKEK
ASWSSLSMDEKVELYRIKFKESFAEMNRGSNEWKTVVGGAMFFIGFTALVIMWQKHYVYGPLPQSF
DKEWVAKQTKRMLDMKVNPIQGLASKWDYEKNEWKK
>COX4-2
MLPRAAWSLVLRKGGGGRRGMHSSEGTTRGGGKMSPYTNCYAQRYYPMPEEPFCTELNAEEQALK
EKEKGSWTQLTHAEKVALYRLQFNETFAEMNRRSNEWKTVMGCVFFFIGFAALVIWWQRVYVFPPK
PITLTDERKAQQLQRMLDMKVNPVQGLASRWDYEKKQWKK
>COX7A-1
MQALRVSQALIRSFSSTARNRFQNRVREKQKLFQEDNDIPLYLKGGIVDNILYRVTMTLCLGGTVYSL
YSLGWASFPRN
>COX7A-2
MLRNLLALRQIGQRTISTASRRHFKNKVPEKQKLFQEDDEIPLYLKGGVADALLYRATMILTVGGTAY
AIYELAVASFPKKQE
>39S ribosomal protein L23, mitochondrial
MARNVVYPLYRLGGPQLRVFRTNFFIQLVRPGVAQPEDTVQFRIPMEMTRVDLRNYLEGIYNVPVAA
VRTRVQHGSNKRRDHRNVRIKKPDYKVAYVQLAHGQTFTFPDLFPEKDESPEGSAADDLYSMLEEER
QQRQSSDPRRGGVPSWFGL
>28S ribosomal protein S10, mitochondrial
MAARTAFGAVCRRLWQGLGNFSVNTSKGNTAKNGGLLLSTNMKWVQFSNLHVDVPKDLTKPVVTI
SDEPDILYKRLSVLVKGHDKAVLDSYEYFAVLAAKELGISIKVHEPPRKIERFTLLQSVHIYKKHRVQY
EMRTLYRCLELEHLTGSTADVYLEYIQRNLPEGVAMEVTKTQLEQLPEHIKEPIWETLSEEKEESKS

>SDHB (binds three Fe-S clusters)
MAAVVALSLRRRLPATTLGGACLQASRGAQTAAATAPRIKKFAIYRWDPDKAGDKPHMQTYEVDLN
KCGPMVLDALIKIKNEVDSTLTFRRSCREGICGSCAMNINGGNTLACTRRIDTNLNKVSKIYPLPHMY
VIKDLVPDLSNFYAQYKSIEPYLKKKDESQEGKQQYLQSIEEREKLDGLYECILCACCSTSCPSYWWN
GDKYLGPAVLMQAYRWMIDSRDDFTEERLAKLQDPFSLYRCHTIMNCTRTCPKGLNPGKAIAEIKKM
MATYKEKKASV
